# Insights into the Activation of a Crustacean G Protein-Coupled Receptor: Evaluation of the Red Pigment-Concentrating Hormone Receptor of the Water Flea *Daphnia pulex* (Dappu-RPCH R)

**DOI:** 10.3390/biom11050710

**Published:** 2021-05-10

**Authors:** Graham E. Jackson, Gerd Gäde

**Affiliations:** 1 Department of Chemistry, University of Cape Town, Private Bag, Rondebosch, Cape Town 7701, South Africa; 2 Department of Biological Sciences, University of Cape Town, Private Bag, Rondebosch, Cape Town 7701, South Africa; gerd.gade@uct.ac.za

**Keywords:** crustacean red pigment-concentrating hormone receptor, Ala replacement scan, molecular docking of mutated ligands, G protein-coupled receptor

## Abstract

The validation of a previously developed model of the interaction between the red pigment-concentrating hormone of *Daphnia pulex* and its cognate receptor (Jackson et al., IJBM 106, 969–978, 2018) was undertaken. Single amino acid replacements, noticeably an Ala scan, of the ligand, Dappu-RPCH, were docked to the receptor, and the binding energies calculated and compared to the one with Dappu-RPCH. As a second step, the same molecules were docked using molecular dynamics (MD) in a 1-palmitoyl-2-oleoyl-sn-glycero-3-phosphocholine (POPC) membrane. Changes in binding energy were compared to previous results on in vitro receptor activation (Marco et al., Sci. Rep. 7, 6851, 2017). Residue scanning and MD simulations both gave comparable results for binding energy. For most mutants, there was a good inverse correlation between in vitro activity and binding. There were, however, exceptions; for example: [Ala4]Dappu-RPCH bound as tightly as the cognate ligand but had little activity. This seeming discrepancy was explained when the MD data were analyzed in detail, showing that, although [Ala4]Dappu-RPCH had multiple interactions with the receptor accounting for the high binding energy, the interacting residues of the receptor were quite different to those of Dappu-RPCH. The MD calculations show clearly that the strong binding affinity of the ligand to the receptor is not sufficient for activation. Interaction of the binding of the ligand to two residues of the receptor, Ser 155 and Gln 237, is also essential. A comparison of our computational results with the experimental results of Marco et al. and comparison with the extensive data on GnRH supports the validity of our Dappu-RPCH R model.

## 1. Introduction

The red pigment-concentrating hormone (RPCH) was the first invertebrate neuropeptide that was fully structurally characterized as a blocked octapeptide (pGlu-Leu-Asn-Phe-Ser-Pro-Gly-Trp amide) in 1972 [1]. It was isolated from the eyestalks of a crustacean, the prawn *Pandalus borealis*, and is hence called Panbo-RPCH. The eyestalks contain the neuroendocrine X-organ (synthesis of neuropeptides) and the neurohemal sinus gland system (for storage and release of neuropeptides); this system is analogous to the well-known vertebrate hypothalamo/hypophyseal system [2]. Functionally, RPCH effects color change in the body integument by concentrating the red pigment granules in epithelial chromatophores. RPCH is a member of the large adipokinetic hormone (AKH)/RPCH family of peptides [3], which is one of three neuropeptide systems of which the mature peptides as well as their cognate G protein-coupled receptors (GPCRs) are structurally similar; the other two systems are called corazonin (CRZ) peptide family and adipokinetic hormone/corazonin-related (ACP) peptide family. All three systems are related to the vertebrate gonadotropin-releasing hormone (GnRH) system and together form a large peptide superfamily [4,5,6]. Panbo-RPCH is apparently restricted to the phylogenetically more advanced crustaceans, i.e., a few orders of the class Malacostraca, but also occurs in some orders of insects [7]. Water fleas of the genus *Daphnia*, which is a more basal crustacean, produce a modified RPCH (Dappu-RPCH: pGlu-Val-Asn-Phe-Ser-Thr-Ser-Trp amide) [8,9,10,11], whereas another crustacean, the fish louse *Argulus siamensis*, is predicted to synthesize yet another modified form (Argsi-RPCH: pGlu-Val-Asn-Phe-Ser-Thr-Lys-Trp amide) [12].

As most neuropeptides signal through GPCRs, this is true for the invertebrate subphylum Crustacea as well. The first cloned crustacean GPCR is from the water flea, *Daphnia pulex* [13], an important model organism for research that centers around ecotoxicology, ecotoxigenomics and evolutionary ecology [14,15,16,17]. The red pigment-concentrating hormone receptor of *D. pulex* (Dappu-RPCH R) belongs to the rhodopsin superfamily of GPCRs and specifically binds the octapeptide crustacean red pigment-concentrating hormone variant, which occurs in *D. pulex* (Dappu-RPCH), but whose function in the water flea is unknown [13].

After the meteoric success of GPCRs as drug targets by pharmaceutical companies for a wide variety of human illnesses and awarding of the Nobel Prize in Chemistry in 2012 for research on GPCRs (www.nobelprize.org), the potential to combat pest insect species via its neuropeptide GPCR complement is being vigorously investigated at present [18,19]. Similar research in Crustacea is in its infancy, but receptor models and docking studies are paramount to develop agonists or antagonists, the final product of which may be peptide mimetics. It can be envisaged that studies on neuropeptide/cognate GPCR systems can be beneficial to a number of practical areas, such as influencing reproduction and growth in aquaculture, to name one example, in which neuropeptides are also involved.

For this reason, we have started experimental work on this first identified Dappu-RPCH R system and treat it as a model for more applied work in future.

As a first step, we have previously used nuclear magnetic resonance and restraint molecular dynamics studies to determine the secondary structure of the agonist, Dappu-RPCH, in a membrane-mimicking environment [20,21]. Although the peptide is quite flexible, two major conformers were established. Both contain β-turns; one conformer has a more open structure, whereas the other conformer’s structure is much tighter [20,21]. Moreover, a 3D model of the Dappu-RPCH R was constructed after the human β2-adrenergic receptor was identified as the top template. Lastly, Dappu-RPCH was docked to its receptor and details of the ligand/receptor interactions were calculated. Although the binding of Dappu-RPCH to its receptor induced significant conformational changes in the ligand, the β-turn was maintained. It is suggested that Dappu-RPCH binds to a pocket formed by both loops and helices with the two termini pointing outwards [20,21].

In the present study, we intend to further validate this model by using specific analogues to the cognate ligand and check their binding to the Dappu-RPCH R according to the data from our previous model. At the same time, a comparison between the different analogues should offer more insight into the mechanism of activation. The analogues have been chosen to represent modifications of the cognate ligand that target the termini (N- and C-terminus) and each amino acid side chain at positions 2 to 8. We also used some other members of the AKH/RPCH family which are analogues of Dappu-RPCH, and we checked a decapeptide to test whether the receptor can also accommodate the binding of larger peptides. The choice of peptides was greatly influenced by a previous study, where these peptides, including Dappu-RPCH, were tested for receptor activation in a mammalian cell-based bioluminescence assay [13].

Docking results are not always reliable, because they only allow for limited movement of the ligand and little movement of the receptor. For this reason, in the current study, the molecular dynamics of the docked structure was also performed in a membrane-mimicking environment.

## 2. Materials and Methods

### 2.1. Residue Scanning

The previously determined structure of Dappu-RPCH docked to Dappu-RPCH R [20,21] was used as the starting construct for residue scanning. This GPCR was built using the 2.4 Å resolution crystal structure of the human β2-adrenergic receptor (PDB id: 2RH1) [22], which was identified as the top template. Residue scanning was performed using the Schrödinger software package [23] with a Prime side-chain prediction that included backbone adjustments. [Ace]Dappu-RPCH and [COOH]Dappu-RPCH were not included in the residue scanning, as these are not mutation options of the Schrödinger software. A non-bonded cut-off of 40 Å was used for the Python Minimizer. As residue scanning calculates relative binding energies, these were converted to absolute binding energies by adding the binding energy of Dappu-RPCH. Table 1 depicts the structures of the peptides that were docked: first, there was the Ala replacements series, in which each residue of the endogenous ligand Dappu-RPCH was successively substituted with an Ala residue; a few bio-analogs were used that occur in nature and have only one substitution compared to Dappu-RPCH, as well as some single Gly replacements plus a decapeptide. In Table 1, we also give the EC_50_ values, as published by Marco et al. [13] for receptor activation in a mammalian-cell-based bioluminescence assay.

The results of docking the various modified peptides to the receptor gave a series of binding energy differences relative to the binding of the endogenous parent Dappu-RPCH ligand.

### 2.2. Molecular Dynamics

In order to mimic the plasma membrane [24], following residue scanning, the docked mutation constructs were inserted into a POPC (1-palmitoyl-2-oleoyl-sn-glycero-3-phosphocholine) membrane using the Schrödinger system builder. SPC water molecules were added, and the construct neutralized by adding Cl^−^ ions. The OPLS3e force field was used, and the system relaxed using the default script. NPT molecular dynamics was performed for 50 ns at 300 K and a pressure of 1.103 bar, using DESMOND [23]. The final 30 ns were analyzed using the ligands Simulation Interaction Diagram tool implemented in Maestro [25]. The binding energy of each snapshot of the trajectory was calculated using the MMGBSA approach, where the generalized Born model and solvent accessibility method are used to elicit free energies for each structure [26]. The thermal mmgbsa.py python script, provided by Schrödinger [23], was used to calculate the ΔG_binding_ for each snapshot. The same process was repeated for other modifications of Dappu-RPCH; replacement of the blocked N-terminal amino acid pyroglutamate with the blocked N-acetyl alanine and a replacement of the amidated C-terminus with the free acid. Dynamics were also performed on the naturally occurring decapeptide, Placa-HrTH, the core of which only differs from Dappu-RPCH at amino acid residue six (Pro vs Thr) and is C-terminally extended by two amino acids. Since the approach in this paper is more extensive than our previous study [21], the calculations were also repeated for Dappu-RPCH.

## 3. Results and Discussion

### 3.1. Residue Scanning

Residue scanning is a quick method of comparing the relative binding energies of two ligands to the same receptor. In this method, the parent ligand residues are systematically mutated to another amino acid—typically alanine or glycine. The change in ligand/receptor free energy of binding, relative to the parent ligand, is then calculated. Because such calculations allow for side chain optimization but for only limited movement of the respective ligand within the binding pocket, they are quickly achieved.

**Figure 1:**
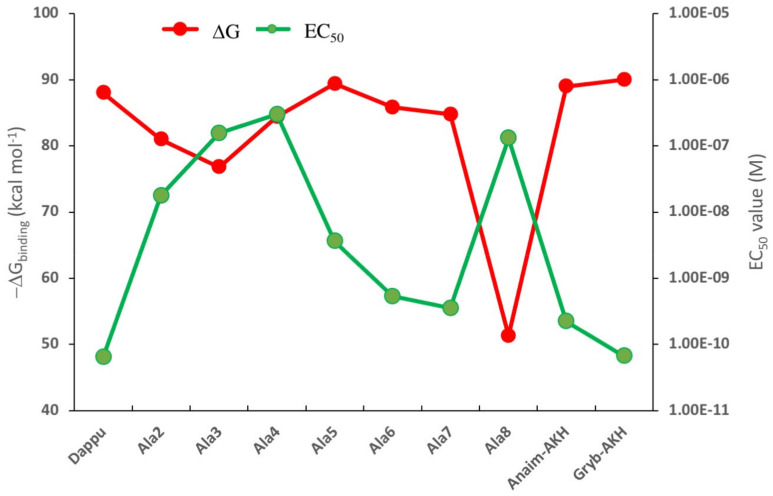
ΔG_binding_ calculated for a series of mutated Dappu-RPCH ligands bound to Dappu-RPCH R, and the EC_50_ values for the same ligands tested on Dappu-RPCH R-expressing cells in an in vitro bioluminescent assay [13]. Only those mutated ligands are shown for which EC_50_ values did exist. Results for [Ace]Dappu-RPCH and [COOH]Dappu-RPCH are not included as they were not residue-scanning, mutation options of the Schrödinger software.

The change in binding energy for each ligand is given in Figure 1, together with the EC_50_ values for those ligands that were previously tested on the Dappu-RPCH R-expressing cells in an in vitro bioluminescent assay [13]. We hypothesized that we would see an inverse relationship between the two parameters, because a high binding energy should result in a low EC_50_ value. This is indeed the case, as depicted in Figure 1. For example, the authors in [13] suggested that Trp8 is important for receptor activation. Thus, the ligand with an Ala8 residue caused very weak receptor activation. Our calculations show exactly the same trend: the ΔG binding energy is drastically lowered (Figure 1). In [13], it was also shown that replacement with Ala at positions 5, 6 and 7 or with Pro at position 6 (=Anaim-AKH) or Gly at position 7 (=Grybi-AKH) resulted in a strong receptor activation. The binding results (Figure 1) support this conclusion in that all these mutations have binding energies comparable to Dappu-RPCH. The only result that does not conform to our hypothesis is the mutation of Ala4 (and, to a certain extent, for Ala3). Experimentally, these two mutations had similar EC_50_ values to Ala8 [13].

### 3.2. Comparison of Residue Scanning and Molecular Dynamics (MD)

**Figure 2:**
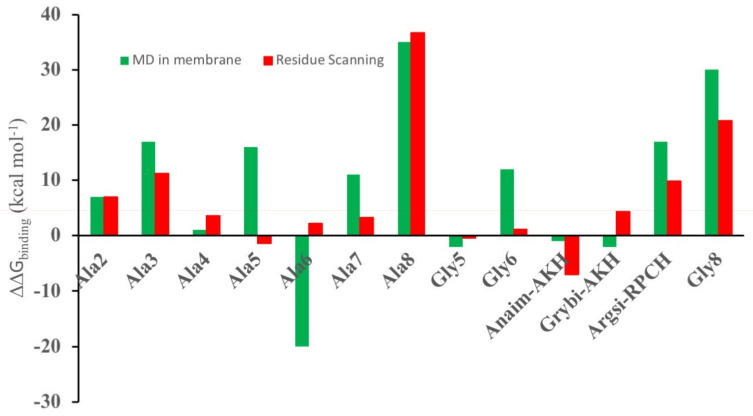
ΔG_binding_ relative to Dappu-RPCH binding to Dappu-RPCHR (ΔG_mutate_ − ΔG_Dappu_). Red bars are from residue Scheme 50. ns simulation of the mutated ligand bound to the receptor in a POPC membrane. Results for [Ace]Dappu-RPCH and [COOH]Dappu-RPCH are not included, as they did not have residue scanning, mutation options of the Schrödinger software.

In practice, both the mutated ligand and the receptor could move to accommodate binding of the ligand. Therefore, a more sophisticated set of calculations were performed: the receptor with the ligand docked using residue scanning was placed in a hydrated POPC membrane and its dynamics simulated for 50 ns. Regular snapshots of the trajectory were then collected and used to calculate the mmgbsa binding energy. In Figure 2, the results of the molecular dynamics calculation and the residue-scanning calculation are compared. With the exception of [Ala5]Dappu-RPCH and [Ala6]Dappu-RPCH, there is agreement between the two calculation methods. This reinforces the use of residue scanning for in silico ligand screening, where rapid evaluation of a large number of ligands is needed.

### 3.3. Molecular Dynamics

**Figure 3:**
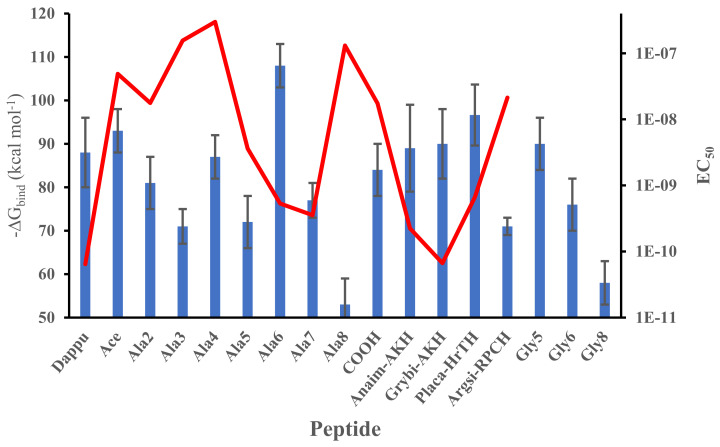
Free energy of binding of mutated Dappu-RPCH to Dappu-RPCHR during the last 30 ns of a 50 ns trajectory in a POPC membrane (blue bar). Error bars are the standard deviation of the mean. EC_50_ values (red line) [13] are not available for some of the peptides.

The full results on the free energy of binding determined from a molecular dynamic simulation of this series of related peptides docked to Dappu-RPCH R are shown in Figure 3. The mean and standard deviation were calculated from the different snapshots collected during the simulation. Many of the peptides have binding energies similar to Dappu-RPCH but some are significantly different, as proven by using the Student’s *t-*test (Table S1 Supplementary Data). [Ace]-, [Ala4]-, [Gly5]-, Grybi-AKH, Anaim-AKH and [COOH]Dappu-RPCH have binding energies which are not significantly different to the parent peptide, Dappu-RPCH. The other peptides have significantly different binding energies. Superimposed on the ΔG graph (Figure 3) is a graph of EC_50_ values [13]. Again, we expect an inverse correlation between ΔG and EC_50_. However, if we compare [Ala6]Dappu-RPCH and [Ala7]Dappu-RPCH: [Ala6]Dappu-RPCH has a ΔG_binding_ of 108 kcal mol^−1^ and [Ala7]Dappu-RPCH only 77 kcal mol^−1^, yet their EC_50_ values are almost the same. [Ala4]Dappu-RPCH has the lowest EC_50_ value but has as high a ΔG_binding_ as Dappu-RPCH. In order to understand the effect of the mutation upon ΔG_binding_, one needs to look at the details of the ligand/receptor interaction, given in the following paragraphs.

#### 3.3.1. [Ala5]Dappu-RPCH

Residue scanning predicts the same binding energy for Dappu-RPCH and [Ala5]-Dappu-RPCH, while the MD calculations predict that Dappu-RPCH binds some 15 kcal mol^−1^ more strongly than [Ala5]Dappu-RPCH. The reason for this is that Ser5 does not contribute to the binding energy and the van der Waals interactions of Ser5 and Ala5 are similar; therefore, residue scanning predicts similar binding energies for the two ligands. With MD, however, it becomes evident that [Ala5]Dappu-RPCH moves in the binding pocket in such a way that Ser155 no longer H-bonds to Asn3. Moreover, the terminal pyroglutamic acid no longer interacts with Gln257. These are two major interactions for Dappu-RPCH to bind tightly and activate its receptor (see later). Since residue scanning does not allow the ligand to move into the binding pocket, this change in interaction is not possible to detect with this calculation method. We conclude from this example that the MD results are more accurate and, hence, they will be used in the discussion from here onwards.

#### 3.3.2. [Ala6]Dappu-RPCH

For [Ala6]Dappu-RPCH, the MD calculation predicts a substantial increase in the binding energy compared to Dappu-RPCH, while residue scanning predicts a decrease in binding energy. In order to explain this discrepancy, one has to look at the details of the ligand/receptor interaction and what factors contribute to the binding energy (Table 2). A major contributor to the MD binding energy is the coulombic energy. ΔG_coul_ is −35 kcal mol^−1^ for [Ala6]Dappu-RPCH but only −11 kcal mol^−1^ for Dappu-RPCH. Since the substitution of threonine by alanine should decrease the coulombic interaction, the increase indicates that the threonine is not interacting with the receptor, and that the ligand has moved into the binding pocket to maximize the coulombic interaction between the receptor and other ligand residues. Another contributing factor is the solvent accessible surface ΔG_solvation_, which is −80 kcal mol^−1^ for [Ala6]Dappu-RPCH, as opposed to −74 kcal mol^−1^ for Dappu-RPCH. This is a major contributor to the total binding energy, which is absent in residue scanning, as there are no explicit water molecules.

#### 3.3.3. Dappu-RPCH

**Figure 4:**
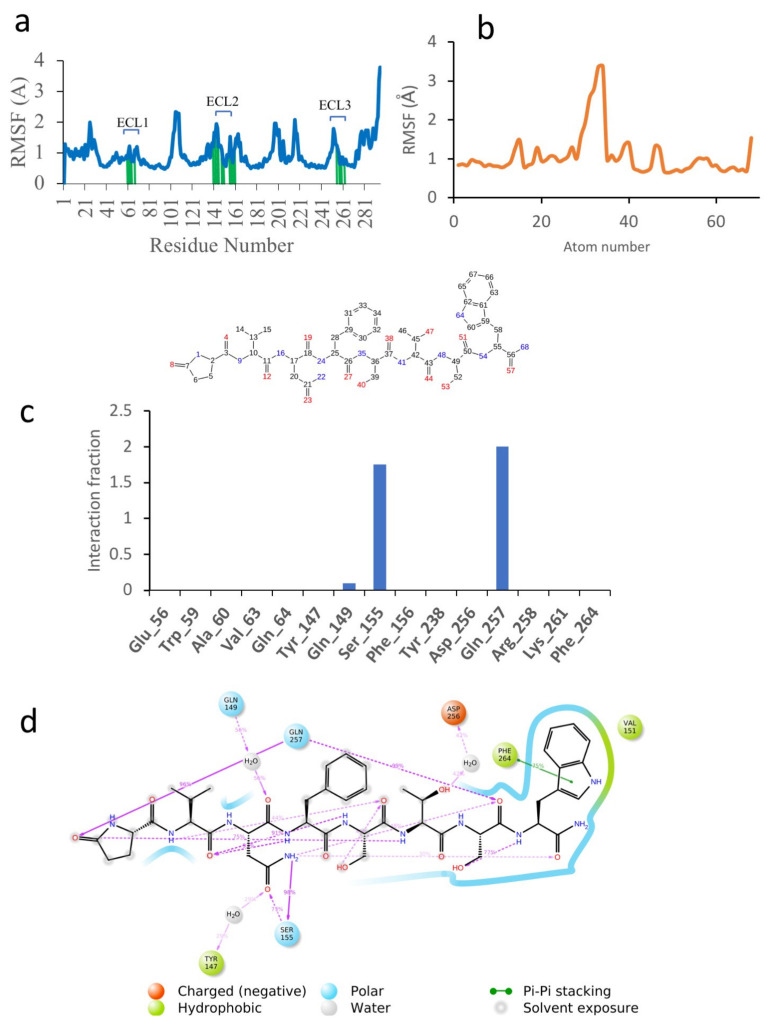
A 50 ns simulation of Dappu-RPCH bound to Dappu-RPCH R in a POPC membrane. (**a**) RMSF of receptor. Protein residues that interact with the ligand are marked with green-colored vertical bars. (**b**) RMSF of ligand. Atom numbers are shown in the schematic view below. (**c**) Receptor/ligand contacts during the simulation. The stacked bar charts are normalized over the course of the trajectory. (**d**) Details of ligand atom interactions with the protein residues. Interactions that occur for more than 30.0% of the simulation time in the selected trajectory are shown. Residues are represented as colored spheres, labelled with the residue name and residue number, and colored according to their properties. The ligand is displayed as a 2D structure. Interactions between the residues and the ligand are drawn as lines, colored by interaction type. The binding pocket is indicated by a line drawn around the ligand, colored by the color of the nearest residue. Solvent exposure is indicated on the ligand atoms, and by the break in the line drawn around the pocket.

In order to gain more insight into the reasons for the different binding energies of the different ligands, the MD results were examined more closely. Figure 4 shows the simulation results for Dappu-RPCH, bound to its receptor. Figure 4a depicts the root mean square fluctuations (RMSF) in the receptor protein C_α_ carbon during the simulation. On this plot, peaks indicate areas of the protein that fluctuate the most during the simulation. Only modest fluctuations were seen and, as expected, the areas of greatest fluctuation correspond to the loop regions. Protein residues that interact with the ligand are marked with green-colored vertical bars. Extra-cellular loop 2, ECL2, which extends over the binding pocket, is a major area of interaction.

The RMSF of the ligand, Dappu-RPCH, is shown in Figure 4b. For a free ligand, the N- and C-termini usually show the largest fluctuation as they are bound only on one side. However, for Dappu-RPCH, the termini are fairly rigid within the receptor-binding site. Most of the movement is at the Phe4 side chain, which moves within a hydrophobic pocket of the receptor.

On the receptor, Ser155 on ECL2 and Gln257 on ECL3 are important contacts (Figure 4c), interacting during the entire simulation. Details of the Dappu-RPCH-receptor contact are shown in Figure 4d. Asn3-NH_d_ H-bonds to Ser155 for 98 % of the simulation, while pGlu1-CO and Ser7-CO, H-bond to Gln257. Additionally, the terminal Trp8 lies in a hydrophobic pocket of the receptor and has a π-π stacking interaction with Phe264 (Phe^7.39^). Here, we see that the turn structure found for Dappu-RPCH [21] is important for receptor binding, as the turn is necessary for Gln257 to simultaneously bind to Asn3-NH_d_ and Ser7-CO.

#### 3.3.4. [Ace]Dappu-RPCH, [COOH]Dappu-RPCH and [Ala8]Dappu-RPCH

Receptor activation data [13] showed that the N- and C-termini protecting groups are important for activation. Molecular modelling, however, shows that the binding energies are not affected.

**Figure 5:**
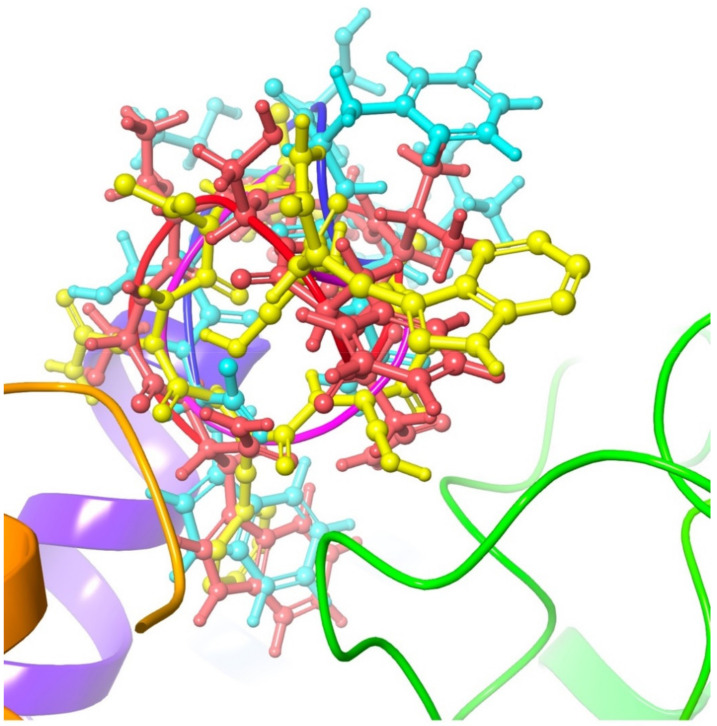
Overlay of Dappu-RPCH (red), Dappu-COOH (blue) and [Ace]Dappu (yellow) in the Dappu-RPCH R receptor binding pocket.

**Figure 6:**
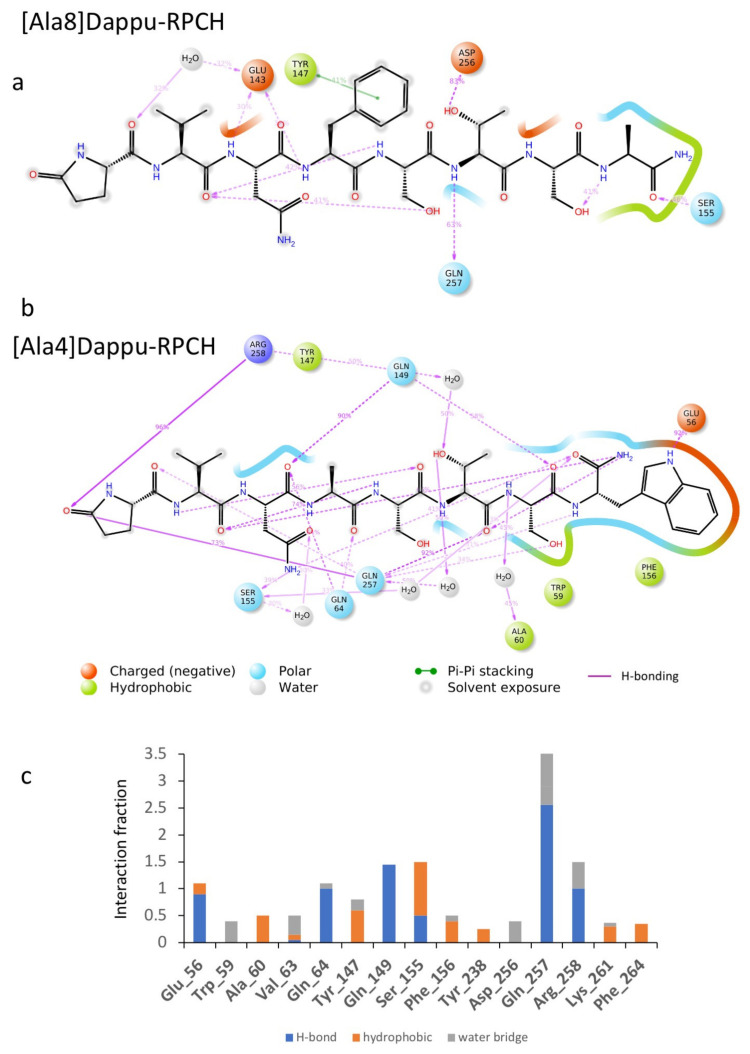
Ligand interaction diagram of (**a**) [Ala8]Dappu-RPCH and (**b**) [Ala4]Dappu-RPCH. (**c**) Interaction fraction of [Ala4]Dappu-RPCH with the receptor averaged over a 50 ns trajectory in a POPC membrane.

ΔG_binding_ of [Ace]Dappu-RPCH is 5 kcal mol^−1^ higher, while [COOH]Dappu-RPCH is 4 kcal mol^−1^ lower than Dappu-RPCH itself. Marco et al. postulated [13] that the decreased activity of the unprotected Dappu-RPCH is due to steric hindrance, conformational changes or ionic interaction with the negatively charged acid group. Details of the energy contribution to ΔG_binding_ (Table 2) show that the negative charge of the COO^−^ group increases the coulombic binding energy. ΔG_coulomb_ for the COOH analogue is −52 kcal mol^−1^, while, for Dappu-RPCH and the ACE analogue, it is −11 and −29 kcal mol^−1^, respectively. All three ligands have very similar lipophilic, covalent, H-bonding and solvation binding energies. However, the van der Waals or steric clashes are different. This supports the suggestion [13] that the negative charge of the carboxylated Dappu-RPCH does play a role. The present results also show that there is not much change in the conformation of the three ligands. This is very evident in Figure 5, where the three ligands were overlaid in the receptor. All three ligands have similar conformations, and Dappu-RPCH and Dappu-COOH are similarly oriented in the binding pocket. [Ace]Dappu, however, is oriented differently: Trp8 is outside the binding pocket. Trp8 is important for receptor activation [13]. The mutation of Trp8 to alanine results in a 35 kCal mol^−1^ decrease in receptor binding. Figure 6a shows the details of the [Ala8]Dappu-RPCH/receptor interaction. The central portion of the ligand still interacts with the receptor, but the C-terminal amide now lies in a hydrophobic pocket and the N-terminal pyroglutamic acid no longer H-bonds to Gln257. This is a clear example to demonstrate that binding energy is not the only criterion important for receptor activation, but also the orientation of the ligand within the binding pocket.

#### 3.3.5. [Ala4]Dappu-RPCH

**Figure 7:**
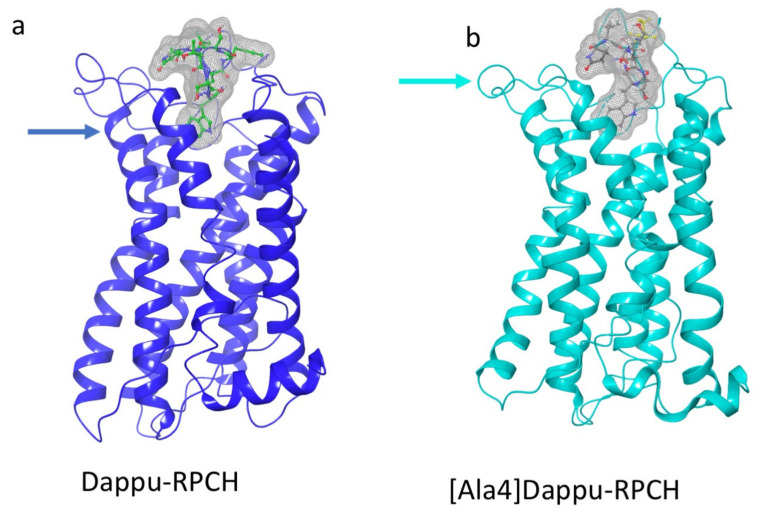
Snapshots taken from a trajectory in a POPC membrane of Dappu-RPCH and [Ala4]Dappu-RPCH bound to Dappu-RPCHR. (**a**) Dappu-RPCH bound to Dappu-RPCH R. (**b**) [Ala4]Dappu-RPCH bound to Dappu-RPCH R. The surface of the ligands is displayed as a mesh. An arrow indicates where the helix closes onto Dappu-RPCH but does not with [Ala4]Dappu-RPCH.

The aromatic group in position 4 is essential for the activation of Dappu-RPCH R (13). This is not explained by the binding energy, as both Dappu-RPCH and [Ala4]Dappu-RPCH have the same binding energy. Figure 6b,c shows that [Ala4]Dappu-RPCH has multiple interactions with the receptor, but that these are different to the interactions of Dappu-RPCH (compare with Figure 4c,d). These multiple interactions account for the high binding energy of [Ala4]Dappu-RPCH. On the other hand, Dappu-RPCH, which has the same overall binding energy, has a much stronger interaction, with only two receptor residues, Ser155 and Gln257 (Figure 4c,d). These two interactions induce the conformational change in the receptor, as can be clearly seen by a MD simulation of the two ligands in a POPC membrane. In Figure 7, the receptor was activated by closing onto Dappu-RPCH, while for [Ala4]Dappu-RPCH, the receptor did not change conformation. The data for [Ala4]Dappu-RPCH suggest that this ligand could be an antagonist. Such an interpretation would have to be experimentally proven and receptor assays performed to confirm it.

#### 3.3.6. Placa-HrTH

**Figure 8:**
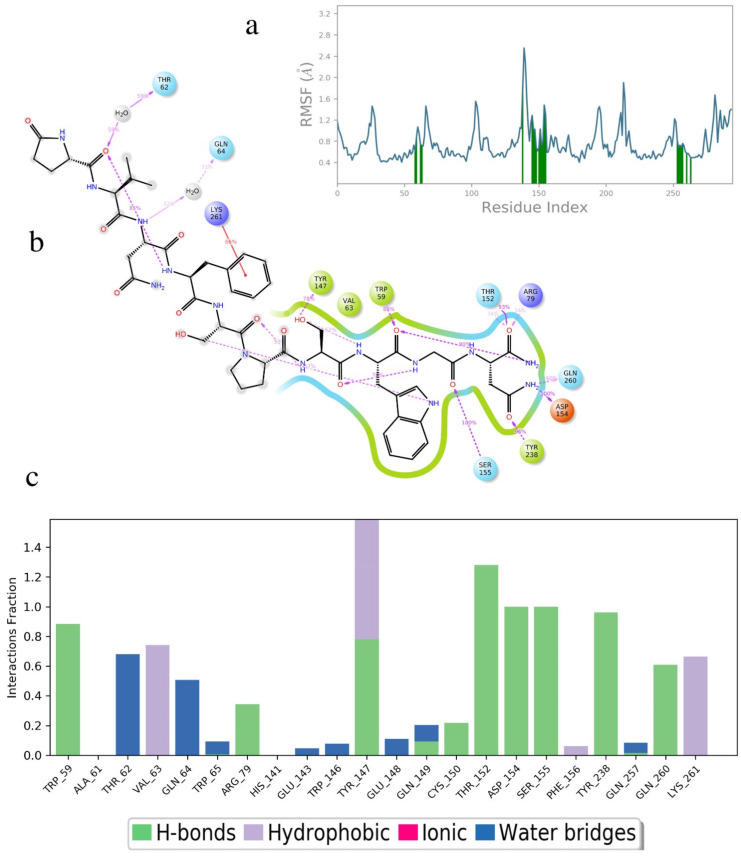
A 50 ns simulation of Placa-HrTH bound to Dappu-RPCH R in a POPC membrane. (**a**) RMSF of receptor. Protein residues that interact with the ligand are marked with green-colored vertical bars. (**b**) Details of ligand atom interactions with the protein residues. Interactions that occur for more than 30.0% of the simulation time in the selected trajectory are shown. Residues are represented as colored spheres, labelled with the residue name and residue number, and colored according to their properties. The ligand is displayed as a 2D structure. Interactions between the residues and the ligand are drawn as lines, colored by interaction type. The binding pocket is indicated by a line drawn around the ligand, colored by the color of the nearest residue. Solvent exposure is indicated on the ligand atoms, and by the break in the line drawn around the pocket. (**c**) Receptor/ligand contacts during. The stacked bar charts are normalized over the course of the trajectory.

Placa-HrTH is a decapeptide first identified in cicadas, where it is a true hypertrehalosemic hormone [27]; thus, increasing the concentration of circulating carbohydrates in the hemolymph of the insect in a fashion similar to the increase in glucose in the blood of vertebrates by the action of glucagon. The binding energy of Placa-HrTH to Dappu-RPCH R is significantly higher (Table 2) than Dappu-RPCH, yet, while it is active, it is less active than Dappu-RPCH in receptor activation [13] Again, in order to explain this discrepancy, the details of the ligand/receptor interactions need to be examined. Placa-HrTH penetrates deeper into the receptor binding pocket than Dappu-RPCH (Figure 8). The terminal amide of Placa-HrTH interacts strongly with Thr152 of ECL2 and Arg79 on TM3. At the same time, Asn10 H-bonds strongly with Asp154 of ECL2 and Tyr238 of TM7 and less strongly (60 %) with Gln260. Ser155 still has a strong interaction with the ligand, but now it is with Gly9-CO rather than Asn3 of Dappu-RPCH. Phe8 still sits in a hydrophobic pocket of the receptor. Placa-HrTH extends out of the receptor binding pocket with residues 1-5, all solvent, exposed. Note that the N-terminus still interacts with the receptor but now does so via a water bridge. Water molecules in the binding pocket are thought to be important for class 1 GPCRs [28].

**Figure 9:**
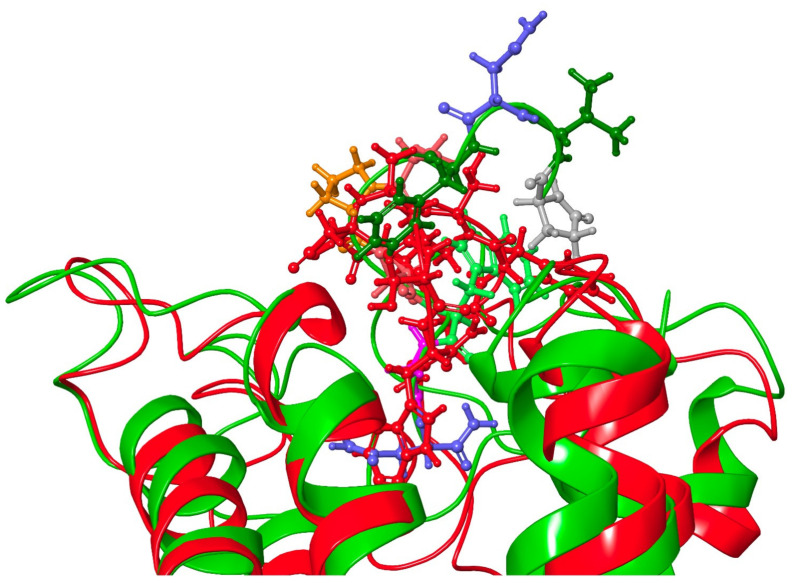
Overlay of Dappu-AKH + Dappu-AKH R (red) and Placa-HrTH + Dappu-AKH R (green). Note the close correspondence of the helical bundles.

[Ala4]Dappu-RPCH had the same binding energy as Dappu-RPCH, yet was inactive in receptor activation studies [13]. By comparing the binding of [Ala4]Dappu-RPCH, which is inactive, and Placa-HrTH, which is active, we can postulate that Ser155 and Gln257 of the receptor are important for activation. Figure 9 shows an overlay of Placa-HrTH bound to Dappu-RPCH R and the conformation of Dappu-RPCH R activated by Dappu-RPCH. The helical bundles overlap remarkably well, indicating that Placa-HrTH has activated the receptor.

#### 3.3.7. Comparison with Gonadotropin Releasing Hormone GnRH

GnRH is the central regulator of mammalian reproduction and, because of its clinical importance, has received extensive study [28]. In order to compare GnRH R and Dappu-RPCH R, the Ballesteros and Weinstein numbering system [29] will be used. Dappu-RPCH R has the same conserved residues as GnRH R in that it has Asn^1.50(16)^, Asn^2.50(35)^, Arg^3.50(97)^, Trp^4.50(122)^, Pro^5.50(177)^, Pro^6.50(237)^ and Pro^7.50(275)^ [28]. However, GnRH H has a DRS (Asp^3.49^-Arg^3.50^-Tyr^3.51^) motif rather than the DRF motif of Dappu-RPCH R. Both receptors have the CWTPY motif on TM6, but GnRH R has a DPxxY motif on TM7 rather than the more common NPxxY motif of Dappu-RPCH. Rhodopsin has an “ionic lock” between Arg^3.50^ and Glu^6.30^, which stabilizes the inactive state of the receptor. In GnRH R and Dappu-RPCH R, Glu^6.30^ is replaced by Arg^6.30^. For GnRH R, mutation studies have shown that both Arg residues are important [27], and so we would predict that the same is true for Dappu-RPCH R. Cvicek et al. [30] have shown that inactive GPCRs have a conserved interaction between Arg^3.50^ and residue 6.37. For both GnRH R and Dappu-RPCH R, this is Thr^6.37^.

Since GnRH is a decapeptide, it is interesting to compare it to the decapeptide, Placa-HrTH, and the octapeptide, Dappu-RPCH, which are both active in Dappu-RPCH R in the receptor activation assay [13]. GnRH has a dominant β-turn conformation similar to Dappu-RPCH. This brings the two termini close together, allowing them to interact with the receptor. Placa-HrTH also has a turn structure but, as it is two residues longer than the endogenous peptide, residues 2-5 form a loop which projects outside the binding pocket. The β-turn of GnRH is stabilized by substitution of the achiral Gly6 by D-amino acids. The turn of Placa-HrTH is stabilized by Pro6, while the turn of Dappu-RPCH is stabilized by H-bonding of pGlu1-CO and Ser7-CO to Gln257. The binding of different peptides to GnRH R showed that the C-terminal of the agonist penetrates the TM cores, while the N-terminus binds to the loop region of the receptor. This same binding pattern is seen for Placa-HrTH and Dappu-RPCH. Dappu-RPCH has a terminal Trp8 which lies between Tyr^6.51(238)^, Phe^7.39(264)^ and Arg^3.32(79)^. Neither Placa-HrTH nor GnRH have this terminal tryptophan, but they do interact with the same receptor residues. For Placa-HrTH, it is the terminal asparagine, while for GnRH, it is Gly10. For GnRH, it was postulated that pGlu1 interacts with Lys^3.32^, but it is uncertain that there is a direct interaction between GnRH and Lys^3.32^. Indeed, our results for Dappu-RPCH and Placa-HrTH show that it is the C-terminus that interacts with residue 3.32 of the receptor. Note for Dappu-RPCH R, Lys^3.32^ is mutated to Arg^3.32^. For Placa-HrTH, pGlu1 has a water-mediated interaction with Thr62, while Dappu-RPCH H-bonds strongly with Gln257. Thus, we can see that there is a large similarity between the three-dimensional structure of GnRH R and Dappu-RPCH R, with the same binding pocket and similar agonist/receptor interactions between the two systems, supporting the view that these two hormonal systems belong to one superfamily, as outlined in the Introduction.

## 4. Conclusions

This MD study of analogues of Dappu-RPCH binding to its receptor has highlighted that several factors are responsible for ligand activity. Firstly, the ligand has to bind to the receptor sufficiently strongly in order to be active. This is shown by the Ala5, 6 and 7 analogues, which all have similar binding energies and activities to Dappu-RPCH. The Ala8 analogue has very low activity and low binding affinity. However, a high binding affinity is not sufficient to guarantee high activity. This is shown by the Ala4 analogue, which has the same binding affinity as Dappu-RPCH but substantially lower activity. Details of the ligand receptor interaction show that binding to Ser155 and Gln257 of the receptor are important for activity. In Dappu-RPCH, Asn3 H-bonds to Ser155. Substitution of this residue by alanine eliminates this interaction, which, in turn, affects activity. Finally, the simulations show that not only is protection of the terminal amine and carboxyl important for prolonging the half-life of the peptides while circulating in the hemolymph, but they also affect the interaction of the peptide with the ligand. In Dappu-RPCH pGlu1-CO H-bonds to Gln257. This interaction is not present with [Ace]Dappu-RPCH. For [COOH]Dappu-RPCH, pGlu1-CO does H-bonds to Gln257, but H-bonding of the carboxylate to Gln149 moves the imidazole of Trp8 out of its hydrophobic pocket into a polar region. The comparison of our computational results with the experimental results of Marco et al. [13] and comparison with the extensive data on GnRH support the validity of our Dappu-RPCH R model.

## Figures and Tables

**Table 1:** Analogues of Dappu-RPCH together with their EC_50_ values tested on the *Daphnia pulex* RPCH receptor according to [13]. Mutated residues are shown in red.

Peptide Name	Peptide Sequence	EC50 Value (M)	
Dappu-RPCH	pQVNFSTSWamide	6.45E-11	
[Ala2]Dappu-RPCH	pQANFSTSWamide	1.77E-08		
[Ala3]Dappu-RPCH	pQVAFSTSWamide	1.56E-07	
[Ala4]Dappu-RPCH	pQVNASTSWamide	2.99E-07	
[Ala5]Dappu-RPCH	pQVNFATSWamide	3.61E-09	
[Ala6]Dappu-RPCH	pQVNFSASWamide	5.32E-10	
[Ala7]Dappu-RPCH	pQVNFSTAWamide	3.55E-10	
[Ala8]Dappu-RPCH	pQVNFSTSAamide	1.31E-07	
[Ace]Dappu-RPCH	[NAc-Ala]-VNFSTSWamide	4.87E-08	
[COOH]Dappu-RPCH	pQVNFSTSW-OH	1.74E-08	
Placa-HrTH	pQVNFSPSWGNamide	6.64E-10	
[Pro6]Dappu-RPCH= Anaim-AKH	pQVNFSPSWamide	2.24E-10	
[Gly7]Dappu-RPCH= Grybi-AKH	pQVNFSTGWamide	6.67E-11	
[Lys7]Dappu-RPCH=Argsi-RPCH	pQVNFSTKWamide	2.13E-8	
[Gly8]Dappu-RPCH	pQVNFSTSGamide	-	
[Gly5]Dappu-RPCH	pQVNFGTSWamide	-	
[Gly6]Dappu-RPCH	pQVNFSGSWamide	-	

**Table 2:** Selected energy contributions to ΔG_binding_ of a series of mutated Dappu-RPCH binding to Dappu-RPCH R. Energy is in kcal mol^−1^ with standard deviations in brackets. Results were calculated using MMGBSA on a trajectory from a 50 ns MD simulation of different peptides bound to Dappu-RPCH R in a hydrated POPC membrane.

Peptide	Total	Coulombic	Hbond	Lipophilic	Solvation (GB)	Solvation (SA)	vdW
Dappu	−88(7)	−11(3)	−1.1(0.9)	−28(1)	26(3)	−74(3)	29(2)
COOH	−85(4)	−52(6)	−1.9(0.7)	−25(2)	62(4)	−68(4)	20(3)
ACE	−94(5)	−29(8)	−2.7(0.3)	−27(3)	35(2)	−71(3)	10(3)
Ala2	−81(4)	−46(4)	−2.6(0.2)	−24(2)	62(2)	−73(4)	10(2)
Ala3	−71(5)	−35(3)	−1.1(0.1)	−24(2)	44(3)	−60(3)	14(4)
Ala4	−87(6)	−44(4)	−3.8(0.8)	−20(3)	47(4)	−60(2)	12(3)
Ala5	−70(5)	−17(4)	−0.9(0.6)	−22(4)	40(1)	−66(3)	31(2)
Ala6	−108(7)	−35(2)	−2.9(0.3)	−24(3)	42(3)	−80(4)	20(3)
Ala7	−77(4)	−21(3)	−1.4(0.5)	−24(4)	35(2)	−64(4)	26(2)
Ala8	−54(5)	−23(2)	−3.1(0.6)	−15(3)	35(2)	−46(2)	13(3)

## Supplementary Materials

The following are available online at https://www.mdpi.com/article/10.3390/biom11050710/s1, Table S1: Student’s *t*-test of null hypothesis of mean ΔG of binding relative to Dappu-RPCH binding to Dappu-RPCH R. The t-critical value is 2.01 for all the peptides except for pEVNFSPSWGN for which it is 1.97.

## References

[1] Fernlund P., Josefsson L. (1972). Crustacean color-change hormone: Amino acid sequence and chemical synthesis. Science.

[2] Gäde G., Herz W., Kirby G.W., Moore R.E., Steglich W., Tamm C.H. (1997). The explosion of structural information on insect neuropeptides. Progress in the Chemistry of Organic Natural Products.

[3] Marco H.G., Gäde G., Saleuddin S., Lange A.B., Orchard I. (2020). Adipokinetic hormone: A hormone for all seasons. Advances in Invertebrate (Neuro)Endocrinology: A Collection of Reviews in the Post- Genomic Era.

[4] Hansen K.K., Stafflinger E., Schneider M., Hauser F., Cazzamali G., Williamson M., Kollmann M., Schachtner J., Grimmelikhuijzen C.J. (2010). Discovery of a novel insect neuropeptide signaling system closely related to the insect adipokinetic hormone and corazonin hormonal systems. J. Biol. Chem..

[5] Roch G.J., Busby E.R., Sherwood N.M. (2011). Evolution of GnRH: Diving deeper. Gen. Comp. Endocrinol..

[6] Gäde G., Šimek P., Marco H.G. (2011). An invertebrate [hydroxyproline]-modified neuropeptide: Further evidence for a close evolutionary relationship between insect adipokinetic hormone and mammalian gonadotropin hormone family. Biochem. Biophys. Res. Commun..

[7] Gäde G., Marco H.G. (2015). The decapod red pigment-concentrating hormone (Panbo-RPCH) is the first identified neuropeptide of the order Plecoptera and is interpreted as homoplastic character state. Gen. Comp. Endocrinol..

[8] Christie A.E., Cashman C.R., Brennan H.R., Ma M., Sousa G.L., Li L., Stemmler E.A., Dickinson P.S. (2008). Identification of putative crustacean neuropeptides using in silico analyses of publicly accessible expressed sequence tags. Gen. Comp. Endocrinol..

[9] Marco H.G., Gäde G. (2010). Biological activity of the predicted red pigment-concentrating hormone of *Daphnia pulex* in a crustacean and an insect. Gen. Comp. Endocrinol..

[10] Christie A.E., McCoole M.D., Harmon S.M., Baer K.N., Lenz P.H. (2011). Genomic analyses of the *Daphnia pulex* peptidome. Gen. Comp. Endocrinol..

[11] Dircksen H., Neupert S., Predel R., Verleyen P., Huybrechts J., Strauss J., Hauser F., Stafflinge E., Schneider M., Grimmelikhuijzen C.J. (2011). Genomics transcriptomics, and peptidomics of *Daphnia pulex* neuropeptides and protein hormones. J. Proteome Res..

[12] Christie A.E. (2014). Peptide discovery in the ectoparasitic crustacean *Argulus siamensis*: Identification of the first neuropeptides from a member of the Branchiura. Gen. Comp. Endocrinol..

[13] Marco H.G., Verlinden H., VandenBroeck J., Gäde G. (2017). Characterisation and pharmacological analysis of a crustacean G protein-coupled receptor: The red pigment-concentrating hormone receptor of *Daphnia pulex*. Sci. Rep..

[14] Soetaert A., Vandenbrouck T., van der Ven K., Maras M., van Remortel P., Blust R., De Coen W.M. (2007). Molecular responses during cadmium-induced stress in *Daphnia magna*: Integration of differential gene expression with higher-level effects. Aquat. Toxicol..

[15] Baun A., Hartmann N.B., Grieger K., Kusk K.O. (2008). Ecotoxicity of engineered nanoparticles to aquatic invertebrates: A brief review and recommendations for future toxicity testing. Ecotoxicology.

[16] Christie A.E., McCoole M.D. (2012). From genes to behavior: Investigations of neurochemical signaling come of age for the model crustacean *Daphnia pulex*. J. Exp. Biol..

[17] Munoz J., Chaturvedi A., De Meester L., Weider L.J. (2016). Characterization of genome-wide SNPs for the water flea *Daphnia pulicaria* generated by genotyping-by-sequencing (GBS). Sci. Rep..

[18] Audsley N., Down R.E. (2015). G protein coupled receptors as targets for next generation pesticides. Insect Biochem. Mol. Biol..

[19] Verlinden H., Vleugels R., Zels S., Dillen S., Lenaerts C., Crabbé K., Spit J., Broeck J.V. (2014). Receptors for neuronal or endocrine signalling molecules as potential targets for the control of insect pests. Adv. Insect Physiol..

[20] Jackson G.E., Pavadai E., Gäde G., Timol Z., Andersen N.H. (2017). Data for the homology modelling of the red pigment-concentrating hormone receptor (Dappu-RPCHR) of the crustacean *Daphnia pulex*, and docking of its cognate agonist (Dappu-RPCH). Data Brief.

[21] Jackson G.E., Pavadai E., Gäde G., Timol Z., Andersen N.H. (2018). Interaction of the red pigment-concentrating hormone of the crustacean *Daphnia pulex*, with its cognate receptor, Dappu-RPCHR: A nuclear magnetic resonance and modeling study. Int. J. Biol. Macromol..

[22] Cherezov V., Rosenbaum D.M., Hanson M.A., Rasmussen S.G.F., Foon S.T., Kobilka T.S., Choi H.J., Kuhn P., Weis W.I., Kobilka B.K. (2007). High-resolution crystal structure of an engineered humanß2-adrenergic G protein-coupled receptor. Science.

[23] (2021). Schrödinger Release 2021-1, Desmond Molecular Dynamics System, D.E. Shaw Research: New York, NY, USA, 2021. Maestro-Desmond Interoperability Tools, Schrödinger, New York, NY, USA. https://www.schrodinger.com/.

[24] Sligar S.G. (2016). Nanodiscs for Structural and Functional Studies of Membrane Proteins. Nat. Struct. Mol. Biol..

[25] (2021). Schrödinger Release 2021-1.

[26] Genheden S., Ryde U. (2015). The MM/PBSA and MM/GBSA methods to estimate ligand-binding affinities. Expert Opin. Drug Discov..

[27] Gäde G., Janssens M.P.E. (1994). Cicadas contain novel members of the AKH/RPCH family peptides with hypertrehalosaemic activity. Biol. Chem. Hoppe Seyler.

[28] Flanagan C., Manilall A. (2017). Gonadotropin-Releasing Hormone (GnRH) Receptor Structure and GnRH Binding. Front. Endocrinol..

[29] Ballesteros J.A., Weinstein W. (1995). Integrated methods for the construction of three-dimensional models and computational probing of structure-function relations in G protein-coupled receptors. Methods Neurosci..

[30] Cvicek V., Goddard W.A., Abrol R. (2016). Structure-based sequence alignment of the transmembrane domains of all human GPCRs: Phylogenetic, structural and functional implications. PLoS Comput. Biol..

