# Supplementary material for: Insights into the Activation of a Crustacean G Protein-Coupled Receptor: Evaluation of the Red Pigment-Concentrating Hormone Receptor of the Water Flea Daphnia pulex (Dappu-RPCH R)
Source: Biomolecules. 2021 May 10;11(5):710. doi: 10.3390/biom11050710 (PMC8151907; doi:10.3390/biom11050710)
Supplement: Supplementary file 1 [file biomolecules-11-00710-s001.zip › biomolecules-1161907-supplementary.pdf]

biomolecules

MDPI

Table S1. Student's t-test of null hypothesis of mean ΔG of binding relative to Dappu-RPCH binding to Dappu-RPCH R. The t-critical value is 2.01 for all the peptides except for pEVNFSPSWGN for which it is 1.97.

Peptide	t-stat	Significance
Ace	1.49	no
Ala2	2.06	yes
Ala3	5.11	yes
Ala4	0.30	no
Ala5	4.71	yes
Ala6	5.96	yes
Ala7	3.31	yes
Ala8	10.01	yes
COOH	1.18	no
Gly5	0.59	no
Gly6	3.53	yes
Anaim-AKH	0.28	no
Grybi-AKH	0.57	no
Argsi-RPCH	5.18	yes
Gly8	8.93	yes
pEVNFSPSWGN	2.62	yes
